# Supplementary material for: Faces in the crowd: Twitter as alternative to protest surveys
Source: PLoS One. 2021 Nov 18;16(11):e0259972. doi: 10.1371/journal.pone.0259972 (PMC8601430; doi:10.1371/journal.pone.0259972)
Supplement: S1 Appendix — (PDF) [file pone.0259972.s001.pdf]

Appendix

Links to the original maps of march routes we used to create buffers and locate individuals to march routes are provided as web-archived links in Table S1. A visualization of our geolocation procedure to a 1km buffer of march routes is provided in Fig S1.

**City**	**Link**
DC	`https://tinyurl.com/DCwmrmp`
Boston	`https://tinyurl.com/Bwmrmp`
Chicago	`https://tinyurl.com/CHIwmrmp`
Denver	`https://tinyurl.com/DEwmrmp`
LA	`https://tinyurl.com/LABAYwmrmp`
NYC	`https://tinyurl.com/NYCwmrmp`
Oakland	`https://tinyurl.com/OAKwmrmp`
Portland	`https://tinyurl.com/PRTwmrmp`
Seattle	`https://tinyurl.com/SEwmrmp`
SF	`https://tinyurl.com/SFwmrmp`

Table S1. Women's March original map archived sources.

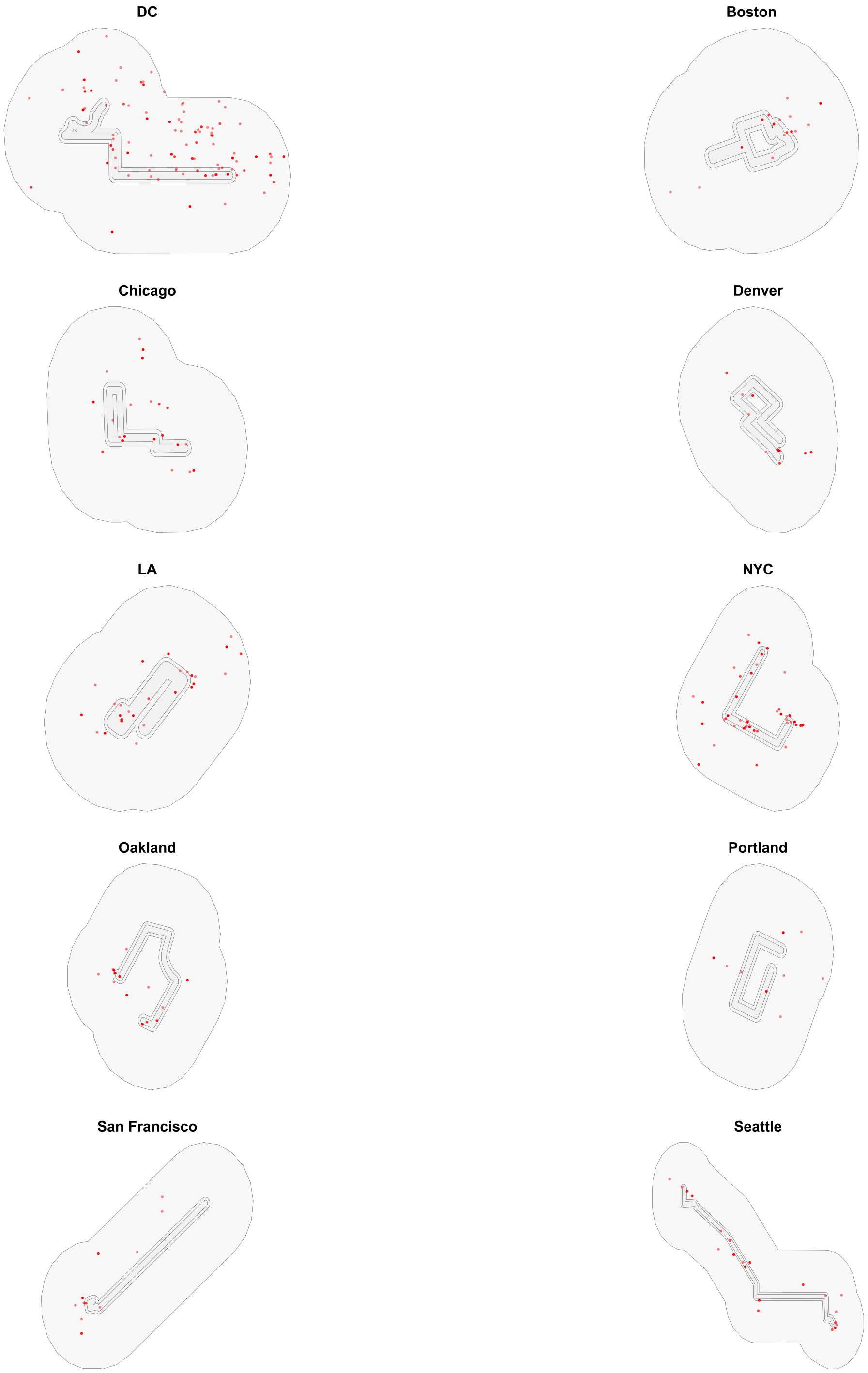

Fig S1. Georeferenced Women's March route maps and geolocated protestor Twitter users in ten US cities.

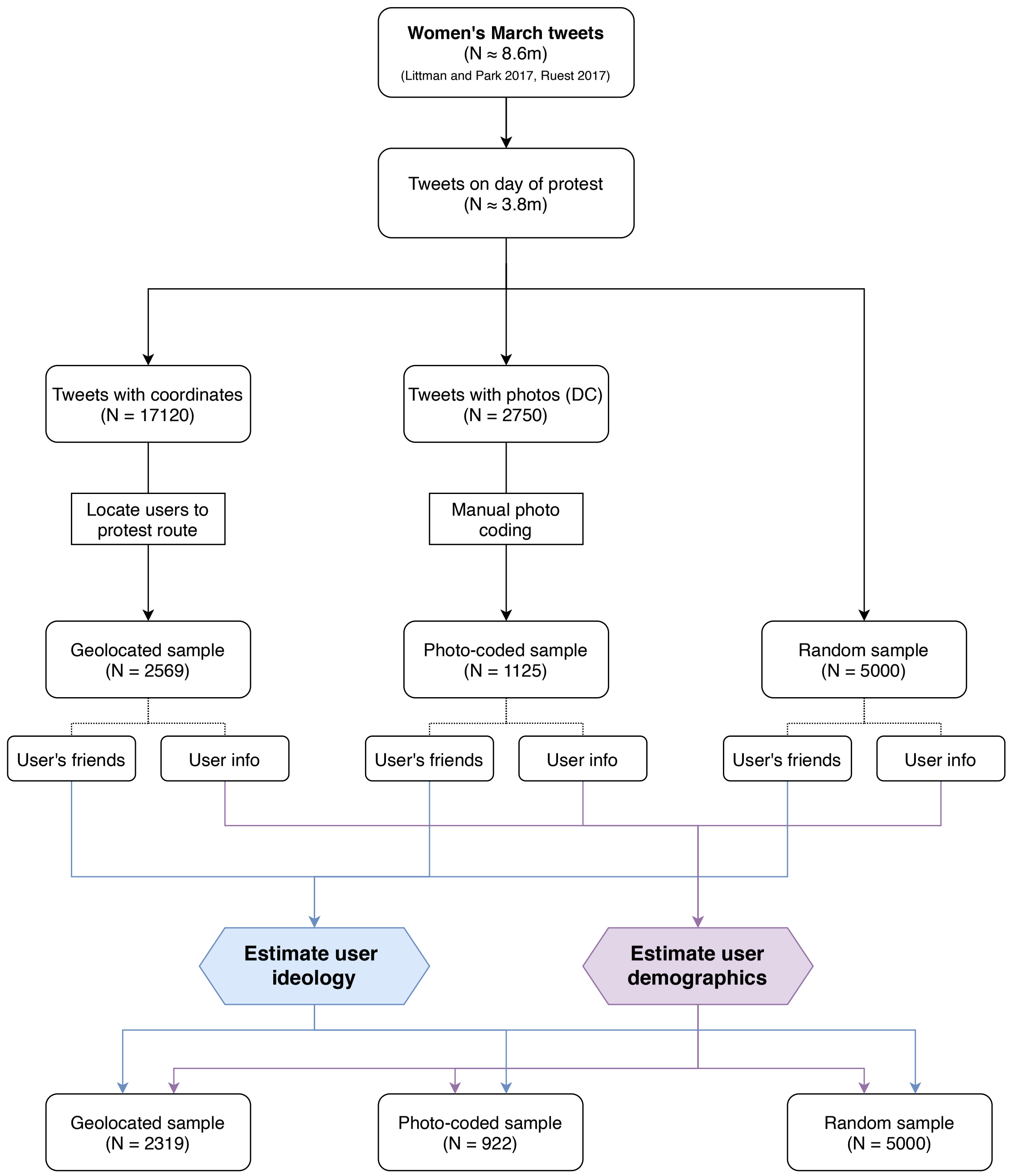

Fig S2. Visualization of data pre-processing and estimation procedure used in calculating crowd ideologies.

Ethical considerations

Twitter data is deemed public by most Institutional Review Boards, including the authors' home institutions. The (updated) guidance provided by the University of Oxford on Internet-Based Research is available at `https://researchsupport.admin.ox.ac.uk/files/bpg06internet-basedresearchpdf`. Given that this is a fast-moving domain and ethical considerations require constant updating, we also consulted, per the advice of the Central University Research Ethics Committee, the guidelines published by the Association of Internet Researchers, available here: `https://aoir.org/reports/ethics3.pdf`. Further, we did not stage any intervention when using these data. As a result, formal ethical review was not strictly required. We nonetheless sought the guidance of our Departmental Research Ethics Committee. The Tweet IDs we used were posted publicly by community organization Documenting the Now in accordance with Twitter's Terms of Service. See `https://catalog.docnow.io/` and `https://developer.twitter.com/en/developer-terms/agreement-and-policy` for further information.

Practitioners have questioned the exemption of public data from ethical review, and have noted tensions in the continued reliance on ethical review guidelines imported from a pre-digital age [1, 2]. A key reason for this skepticism derives from adherence to norms of contextual integrity [3]. Using social media data for academic research removes the data from the original context of its intended reception, notwithstanding its public status [4]. We believe our study *does* constitute a violation of contextual integrity, according to the nine principles set out by [3] and extended to social media research by [4]. We have removed these tweets from the original context of their dissemination and likely intended audience [5, 6]. What is more, we have processed these data in a way that facilitates the connection of an individual with a particular—and potentially sensitive—form of political behaviour. If users become disinclined to share information on associational activities such as protest as a result, there is a small chance that studies such as ours could harm civil society.

Based on these considerations, we determined 1) to elaborate an anonymization procedure

for each analysis script to minimize the authors' exposure to identifying information; 2) that user-specific information on protestors—i.e., individuals we located to protest vicinities—would be stored in encrypted folders; and 3) that we will not include the identifiable geocoded or photo-coded protestor data subset in public replication files. While our approach does not eliminate the concerns we outline, we believe it helps significantly to mitigate them. In taking these measures, we follow best practice advice in the literature by aiming to reduce the likelihood that sensitive information will be traced back to individual users, by limiting researchers' own exposure to individual account information, and by taking responsibility for the custodianship of processed data [7, 8].

Twitter image coding criteria

To classify images in our Twitter dataset, we formulated a coding framework that followed a set of qualitative criteria. These criteria sought accurately to capture protestors on the ground by excluding: 1) photos not of protest; 2) photos accompanied by text indicating news reporting; and 3) photos that could be stock images. We provide the coding criteria used by both authors below.

Coding Criteria

We code as DC marchers tweets that include a photo, where:

1. The photo is taken from within the crowd, or on the way to the march
2. The caption accompanying the photo indicates that the user:
    (a) is at the march
    (b) is on the way to the march
    (c) is about to leave to go to the march
    (d) has just returned from the march
3. The text acompanying the photo describes the march, or uses popular hashtags
4. The text does not refer to a march outside of Washington, DC
5. The text and photo both indicate participation in the protest
6. The tweet does not indicate news reporting from official news media, and is not just purely descriptive and informational
7. The tweet is not just focusing on signs, but:
    (a) has to be accompanied by text indicating participation in the march, or

   (b) taken from within the crowd, or
   (c) has to be accompanied by text indicating that the user spotted this sign at the protest (e.g. one of my favourite signs I saw today)

8. The image is not curated or photoshopped
9. The photo is not of just children at march
10. The image is a selfie but includes text indicating that individuals are known to the tweeter and together at march.

Missingness

Both estimates of ideological and crowd composition suffer from some degree of missingness. Estimation of ideology scores relies on the following network of a Twitter user. If a user follows no elite accounts, their ideology score cannot be computed. This is the case for 111 observations (4.3%) in the Geo-located and 199 (17.7%) of the Photo-coded sample. In 7 further cases (5 for the Geolocated and 2 for the Photo-coded samples) no finite ideology estimate could be computed, so that the final missingness across both samples is 106 and 201. Estimates of demographic composition rely only on a user's profile information, and should in principle not include any missing data. Still, compiling errors and insufficient profile information results in 5.8% and 4.4% missing demographic data for the Geo-located and Photo-coded samples respectively. The considerable difference in missingness in our ideology estimates between the Geo-located and Photo-coded samples is due to differences in the timing of both estimations: the Photo-coded sample was added in the last stages of the writing process, to benchmark the findings of the geo-located sample against. Differences in missingness thus likely reflect the share of additional users who have made their profile private, deleted their Twitter profile, or had their account removed.

	**Geo-located**		**Photo-coded**	
	N	%	N	%
Ideology Score	116	4.5%	201	17.9%
Gender	148	5.8%	49	4.4%
Age Group	148	5.8%	49	4.4%
**Sample size (incl. missing)**	2519		1125	
**Sample size (excl. missing)**	2319		922	

Table S2. Missingness for ideology and demographic estimates for photo-coded and geolocated samples of Twitter protestors.

Following elite accounts

For our Geolocated and Photocoded samples, as well as our Random sample we also provide below descriptive statistics on the number of elite accounts they follow (see Fig S3). If a user follows only a small number of elite accounts, their ideology score will be estimated with larger error. Nonetheless, the mean and median number of elite accounts followed is reasonable: 47 and 31 respectively for our Geolocated/Photocoded users; 28 and 12 for our Random sample. If we understand the N. of elite follows as a proxy for political interest, the larger number of elite accounts followed by our protestors on the ground is another indication that hashtag sampling will return samples that also differ on this basic characteristic.

To assess whether the distributions we report are driven by users who follow only few elites (and whose ideological position are thus more imprecisely estimated) we repeat the analysis but only include users who follow at least 5 elite accounts. Fig S4 reveals that this has no impact on the ideological distribution of our three samples. In fact, the only sample whose distribution somewhat changes is the Random sample, which is likely due to us excluding users with a lower level of political engagement.

Further sample characteristics

Sampling on hashtag alone, as previous research has done, risks including within the sample both commentators and opponents, as well as individuals who are only mobilizing online. By geolocating our users in our first "geolocated" sampling approach, we effectively remove the possibility of including protestors who are only active online as we are able to locate them to the route of the march. This approach, however, does not exclude the possibility that users in our sample geolocated to in or around the protest route might be either commentators (e.g., journalists, bloggers) or opponents. To scrutinize this, we first calculated the percentage of our geolocated samples in each of our ten cities who were "verified" users. The criteria for user "verification" has changed over time: it was initially used as a means to verify that accounts

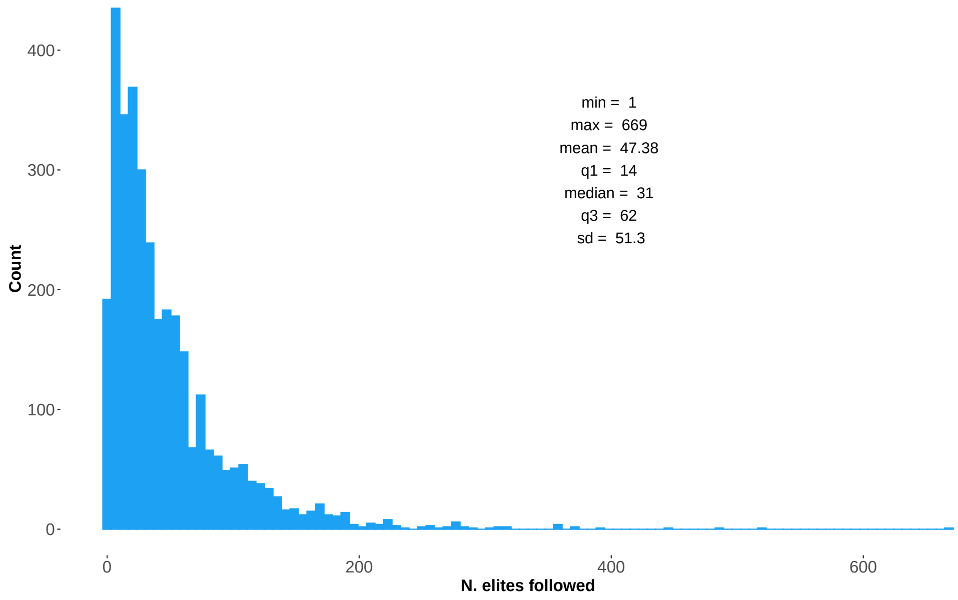

(a) Geolocated/Photo-coded samples

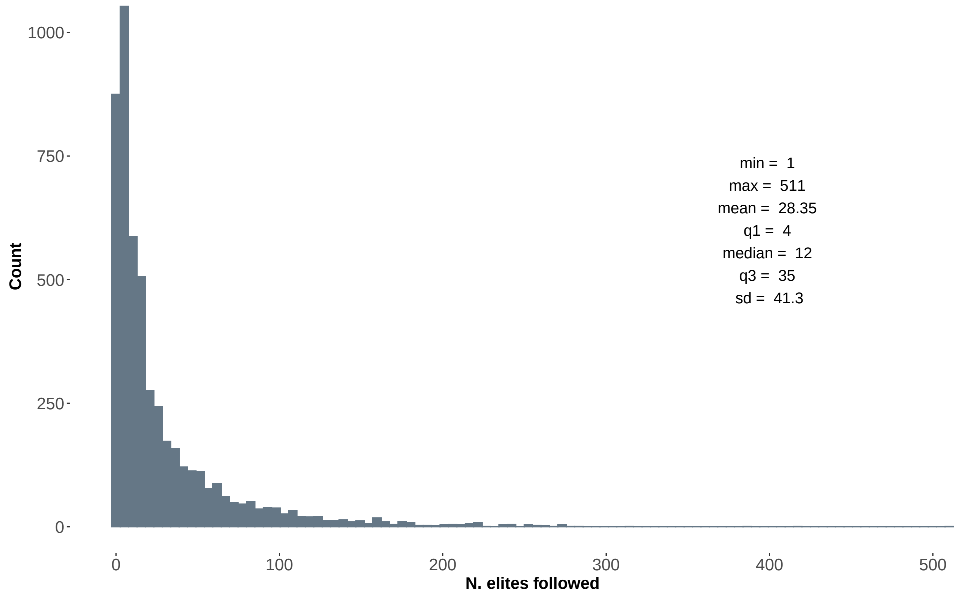

(b) Random sample

Fig S3. Number of elite accounts followed in Geolocated/Photo-coded and Random user samples

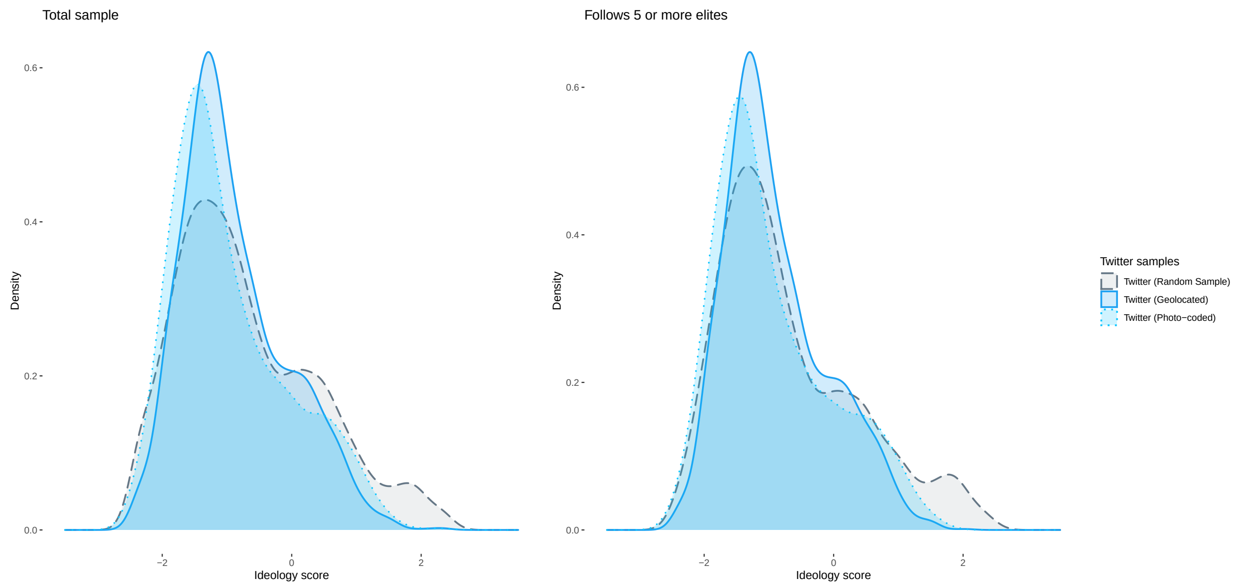

Fig S4. Comparison of ideology distributions of complete sample with ideology distribution of samples where users who followed less than 5 elites were excluded

claiming to be celebrities or other individuals of public notoriety were genuine. It is also often granted to news organizations and journalists. Users granted "verified" status have a blue check mark against their user name. Indeed, the new verification guidelines (see: `https://help.twitter.com/en/managing-your-account/twitter-verified-accounts`) include "News organizations and journalists" as a category of user eligible for verification.

Table S3 displays the percentage of users in each of our samples who are verified users. These range from 0-7%.

N. verified	N. non-verified	% verified	City
0.00	83.00	0.00	Seattle
66.00	926.00	6.65	DC
14.00	417.00	3.25	NYC
44.00	544.00	7.48	LA
0.00	134.00	0.00	Boston
2.00	54.00	3.57	Portland
2.00	36.00	5.26	San Fran.
3.00	87.00	3.33	Oakland
2.00	89.00	2.20	Chicago
2.00	64.00	3.03	Denver

Table S3. Verified Twitter users by protest sample

We also coded the same random sample, on the basis of tweet text and user description, for potential oppositional content. One tweet (.2%) contained hostile sentiment toward the protest (though also mentioned gun rights as a women's rights issue, indicating the individual may have been a protestor but with an ideological outlook not congruent with the majority of other protestors).

In summary, while our geocoding procedure does not entirely filter out commentators on the ground, the size of any bias induced by their inclusion will be minimal. In other contexts, for example with smaller protests, the size of this bias will be non-negligible, meaning that other techniques such as the photo-coding procedure we outline may be preferable. It is also relatively straightforward to identify commentator accounts from their number of followers, verification status, user description, and tweet content.

As for opponents, this appears to be less of a concern, at least in the case of the Women's March. For more contentious protests, or protests that attract counter protests, it will be a larger task to parse supporters and opponents. In this case, filtering by geolocation and then manually coding a random subsample may be the best approach. To scale this approach, automated classification techniques may also be used to label users as opponents or supporters based on user-level and tweet-level characteristics (see e.g., [9]).

References

1. Chancellor S, Birnbaum ML, Caine ED, Silenzio VMB, De Choudhury M. A Taxonomy of Ethical Tensions in Inferring Mental Health States from Social Media. In: Proceedings of the Conference on Fairness, Accountability, and Transparency - FAT* '19. Atlanta, GA, USA: ACM Press; 2019. p. 79–88.

2. Conway M. Ethical Issues in Using Twitter for Public Health Surveillance and Research: Developing a Taxonomy of Ethical Concepts From the Research Literature. Journal of Medical Internet Research. 2014;16(12):e290. doi:10.2196/jmir.3617.

3. Nissenbaum H. Privacy as Contextual Integrity. Washington Law Review. 2004;79:41.

4. Zimmer M. Addressing Conceptual Gaps in Big Data Research Ethics: An Application of Contextual Integrity. Social Media + Society. 2018;4(2):205630511876830. doi:10.1177/2056305118768300.

5. Williams ML, Burnap P, Sloan L. Towards an Ethical Framework for Publishing Twitter Data in Social Research: Taking into Account Users' Views, Online Context and Algorithmic Estimation. Sociology. 2017;51(6):1149–1168. doi:10.1177/0038038517708140.

6. boyd d, Crawford K. Critical Questions for Big Data: Provocations for a Cultural, Technological, and Scholarly Phenomenon. Information, Communication & Society. 2012;15(5):662–679. doi:10.1080/1369118X.2012.678878.

7. Clark K, Duckham M, Guillemin M, Hunter A, McVernon J, O'Keefe C, et al. Advancing the Ethical Use of Digital Data in Human Research: Challenges and Strategies to Promote Ethical Practice. Ethics and Information Technology. 2019;21(1):59–73. doi:10.1007/s10676-018-9490-4.

8. shakti franzke a, Bechmann A, Zimmer M, Ess C, AoIR. Internet Research: Ethical Guidelines 3.0. Association of Internet Researchers; 2020.

9. Rafail P. Nonprobability Sampling and Twitter: Strategies for Semibounded and Bounded Populations. Social Science Computer Review. 2018;36(2):195–211.
